# Explanatory model of the psychosocial variables related to the social acceptance of a uranium mine project in northwest Spain

**DOI:** 10.3389/fpsyg.2023.1134499

**Published:** 2023-05-23

**Authors:** Gonzalo Sánchez-Tabernero, Antonio R. Hidalgo-Muñoz, José Ignacio Galán, Carmen Tabernero

**Affiliations:** ^1^Faculty of Economics and Business, University of Salamanca, Salamanca, Spain; ^2^Faculty of Psychology, University of Salamanca, Salamanca, Spain; ^3^Instituto de Neurociencias de Castilla y León (INCYL), University of Salamanca, Salamanca, Spain

**Keywords:** social conflict, uranium mine, acceptance or rejection, explanatory model, environmental beliefs, risk perception

## Abstract

**Introduction:**

The demographic growth and the development of the welfare system have been accompanied by an important social dilemma between preserving nature or promoting energy development by assuming the benefits and risks of both proposals. This research attempts to address this social dilemma by analyzing the psychosocial factors that influence the acceptance or rejection of a new uranium mining development and exploitation project. The main objective was to test an explanatory theoretical model of uranium mining project acceptance, based on the interrelation of sociodemographic variables (e.g., age, gender, economic and educational situation, and level of knowledge about uranium energy) and cognitive variables (e.g., environmental beliefs, risk, and benefit perceptions), along with the activation of an emotional balance in response to the proposal of constructing a uranium mine.

**Method:**

Three hundred seventy-one individuals responded to the questionnaire about the variables included in the model.

**Results:**

The results showed that older participants showed lower levels of agreement with the mining proposal people, while women and those with greater knowledge of nuclear energy perceived greater risks and had a more negative emotional balance. The proposed explanatory model based on sociodemographic, cognitive, and affective variables showed good fit indices for explaining the assessment of the uranium mine. Thus, age, level of knowledge, risks and benefits, and emotional balance had a direct effect on the acceptance of the mine. Likewise, emotional balance showed a partial mediation effect between the relationships existing between the perception of benefits and risks and the acceptance of the mining proposal.

**Discussion:**

The results are discussed based on the consideration of analyzing sociodemographic, cognitive, and affective variables to understand potential conflicts in communities affected by energy projects.

## 1. Introduction

The concept of sustainability encompasses different dimensions and aims to find the appropriate balance between preserving the natural environment and the development of human activity, thus considering a balance between biodiversity, economy, culture, heritage, and identity roots (Teerikangas et al., 2021). The growth of the population, combined with the development of the welfare society, has led to an increase in investment in research, development, and eco-innovation in order to ensure the necessary resources to meet the population’s various needs, including energy. However, in the face of this approach, Smil’s (2018) asserts that the negative aspects of advances in the development of new energy sources must also be valued, including the deterioration of air quality, inequality in access to energy or energy poverty, or the development of large monopolies that can corrupt governments. Faced with this social dilemma of development versus conservation, it is worth mentioning Sustainable Development Goal 7, which values the importance of a clean, affordable, and modern energy system worldwide (Wang et al., 2022). Thus, in recent decades, numerous alternative energy sources have been explored in an attempt to minimize the negative consequences of pollution, deforestation, and the exploitation of fossil fuels (Afshan et al., 2022).

Therefore, the exploration of new sources of energy has always been accompanied by a debate between development and conservation (Lindahl et al., 2018); for Smil (2018), it is a debate between the positive and negative aspects of development. In this sense, Firestone and Kempton (2007) argued that technological interventions in the environment require a confrontation between local communities, developers, and authorities who support technological intervention. In an attempt to avoid such confrontation, other sources of energy have recently been explored that are further away from densely populated areas. For example, there has been progress in extracting oil and gas in coastal areas (known as offshore drilling). However, in some cases, these interventions also provoke social rejection, as happened in the drilling project off the coasts of the Canary Islands, where, despite having the support of the central administration, the social rejection of the local population ultimately forced the drilling company to abandon the project (Ruiz et al., 2018). Thus, proposals for intervention in nature in search of new sources of energy have often been accompanied by the rejection of the population most directly affected by the construction of such infrastructure (Batel and Rudolph, 2021).

The weight of negative attitudes from the population towards some energy interventions, such as offshore oil and gas drilling, which lead to the effective rejection of different energy projects, has been pointed out in recent research works (Chen and Martens, 2021). In fact, numerous studies (see Upham et al., 2015) have highlighted the role of community acceptance of a proposed energy project as a key factor for the successful execution of such projects by governments. Upham et al. (2015) pointed out that public opinion, perceptions, acceptance, attitudes, behaviors, values, and related practices have become relevant factors for governments, the energy industry, and researchers in environmental and behavioral disciplines. Thus, some research studies (Upham et al., 2015; Chen and Martens, 2021) show that some projects are executed with the approval of affected local communities, while others generate greater rejection, all without knowing the crucial psychosocial factors involved in acceptance. These results highlight the role that populations play in the acceptance or rejection of different energy proposals, hence the emergence of a new concept called “energy citizenship,” based on the democratic right of citizens to engage and participate in decision-making (Devine-Wright, 2007; Brondi et al., 2016).

As mentioned, in the field of energy, social acceptance of technology or innovations in renewable energy is increasingly considered one of the many issues that determine the success of the implementation of new developments and policies (Wüstenhagen et al., 2007). But before advancing with the aim of this research, it seems necessary to emphasize the concept of social acceptance, as Dessi et al. (2022) state, “acceptability” refers to the characteristics that favor a behavioral response for or against; “acceptance” is the behavior that accepts and promotes the use of technology, while “adoption” is the decision-making process (analysis, selection, purchase, and commitment to use) until the technology is used. Social acceptance is one of the key aspects of policy development in the field of energy technologies and, for this reason, a considerable number of sociological and psychological studies focus on analyzing the determinants of social acceptance of a wide variety of energy interventions (Cohen et al., 2014; Batel, 2020). For Devine-Wright and Wiersma (2020), social acceptance is a multidimensional concept in which political, social, economic, and community aspects are interrelated. Thus, acceptance has been defined as a positive attitude towards a specific fact that manifests itself in the form of a supportive, consenting, or authoritative opinion or behavior (Kraeusel and Möst, 2012). Therefore, following Wolsink (2018), the term acceptance aims to encompass both opinions and actions that are relevant to the degree to which the energy innovation project would be accepted or not by the community.

Using a social cognitive theoretical framework, Mendoza-Denton et al. (2020) elaborated a dynamic system of interaction between cognitive and affective variables to explain the processing of information and the behavior of individuals or communities in specific situations. In this line, Baran et al. (2023) use a social cognitive theoretical framework from which the individual processes information, activates an emotional state that explains decision-making or behavior towards an energy innovation. Similarly, Carlson et al. (2020) explain how environmental dispositions are associated with both environmental attitudes and beliefs and the emotional activation that occurs in response to images of climate change with positive or negative valence. Based on the interrelationship between social, cognitive, and affective variables, Huijts et al. (2012) developed a theoretical model to explain the acceptance of energy projects. According to Huijts et al. (2012), some of the population might focus on perceived risks (both environmental and economic, weighing the impact on the current socio-economic system or tourism in the area), and the other part might mainly value the social and economic benefits for the affected area (increased job opportunities, improvements in the communication network and infrastructure, or increased population). Although Huijts et al. (2012) limited the model to psychological variables, they acknowledge that sociodemographic variables could be determinants to explain acceptance. In the present research, a socio-cognitive-affective theoretical framework is used as a starting point to analyze the interrelationship of sociodemographic, cognitive, and affective variables in relation to the acceptance of an energy proposal.

Although *sociodemographic variables* have been shown to have a rather modest explanatory weight in some behaviors, some studies have found a direct relationship between educational and socioeconomic level and acceptance of technological proposals (Devine-Wright and Batel, 2013). For example, education and economic level were related to the acceptance of wind farm implementation (Devine-Wright and Batel, 2013) and technological intervention projects (Brannstrom et al., 2022). Thus, Brannstrom et al. (2022) found that in local communities with low education levels and few employment opportunities, acceptance of an energy proposal increases as the perception of greater economic benefits for the area increases, and therefore, perceived risk decreases. Regarding contextual characteristics, Perko et al. (2015) found that the level of knowledge about nuclear energy is related to negative emotions and attitudes towards this type of energy, and therefore greater perception of risk, such as radiological risks. In relation to the acceptance of the construction of uranium mines, Bjørst (2016) analyzed the role of personal variables that affect the debate between “saving” or “destroying” the local community. In contrast to other types of energy projects, Pedersen and Johansson (2012) compared emotional reactions to a wind farm and a uranium mine, and while they did not find gender differences in acceptance of a wind farm, they did find differences in acceptance of a uranium mine, with a more negative emotional state in women than in men. In addition, older citizens also perceived the uranium mine more negatively and threateningly than younger ones. Therefore, based on these results, a relationship is expected to be found between sociodemographic variables and the level of knowledge, both with environmental beliefs and perceived risks/benefits, as well as with the generated emotional state and acceptance of the energy proposal.

According to the theory of planned behavior proposed by Ajzen (2001), beliefs about specific people or objects influence attitudes, which in turn influence behavioral intention. In this sense, research has shown that *pro-environmental beliefs* are associated with positive public attitudes towards renewable energies and the resulting acceptance or rejection of energy projects (Eurobarometer, 2005; Tendero, 2021). In fact, citizens’ attitudes can differ depending on the type of energy; for example, with regard to oil drilling, several studies have found that most local communities have clearly negative attitudes because they consider drilling to be harmful to the environment and health (Binder and Boldero, 2012). Regarding nuclear energy (Jones et al., 2016), clear differences have been observed in attitudes between different countries. While de Groot et al. (2020) compared the level of risk perception between two types of energy, gas and nuclear, and in both cases, they found that perceived risk leads to rejection of the proposed energy. Beliefs are directly associated with risk perception (or gain perception), which is an essential process for the acceptance of a particular technological project. Thus, perceived benefits and risks have a powerful effect on emotional reactions and the level of acceptance (de Groot et al., 2020). In this sense, in the present research, we find it interesting to analyze the relationship between environmental beliefs, risk and benefit perception associated with the development of the uranium mine, both with the emotional state it generates in citizens and with the possible acceptance of the mining project.

In general, changes in the natural environment can provoke different *affective reactions* that influence social conflict over the impact on nature caused by the exploitation of different energy sources (Ruiz et al., 2018). Previous studies have found that the emotions triggered by energy technologies depend on the personal assessment of the level of threat or opportunity they pose for daily life. In the case of nuclear energy, some studies have shown how the perception of risk and threat posed by this type of energy is associated with rejection of both the creation of nuclear power plants and the radioactive waste associated with uranium (Pidgeon et al., 2008), so opposition to this type of energy is driven by fear and threat. Automatic emotional responses of fear or anxiety can be triggered by knowledge of risks or accidents in other facilities, leading to a greater perception of risk (Venables et al., 2012). Thus, the relationship between perceived risk and the benefit of constructing a uranium mine can determine acceptance or rejection based on the emotional balance activated in the affected population, so emotional balance would play a mediating role in the relationship between perceived risk or benefit and mine acceptance.

After reviewing the interrelationships between sociodemographic variables (i.e., Devine-Wright and Batel, 2013; Bjørst, 2016), cognitive variables (i.e., Devine-Wright, 2009, 2011; Bronfman et al., 2012; Upham et al., 2015), and emotional variables (i.e., Van der Horst, 2007; Devine-Wright, 2011; Huijts et al., 2012; Ruiz et al., 2018) regarding the acceptance or rejection of energy projects from a cognitive, emotional, and personality dynamics approach (Mendoza-Denton et al., 2020), Figure 1 presents a theoretical explanatory model of energy project acceptance or rejection. Thus, the main objective of this study was to analyze the explanatory role of some sociodemographic, cognitive, and emotional variables on the level of acceptance of a uranium mine development project. The specific hypotheses to be tested are as follows:

**Figure 1:**
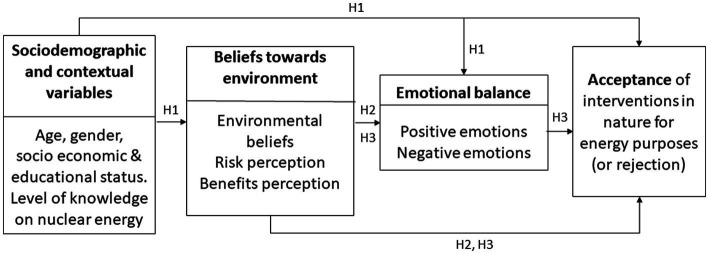
Theoretical explanatory model proposed for the acceptance or rejection of proposals for intervention in nature for energy purposes.

*Hypothesis 1*. Sociodemographic characteristics and level of knowledge about uranium energy will be related to both perceived risks and benefits, as well as to the emotions triggered and acceptance of the proposal. Thus, older people and those with lower socioeconomic and educational levels will perceive greater risks and fewer benefits and, therefore, show lower acceptance of the energy proposal. Likewise, based on the aforementioned studies, we expect to find gender differences whereby women will have stronger environmental beliefs and perceive greater risk regarding the construction of uranium mines than men, leading to greater emotional balance activation and consequent rejection of the mining proposal.

*Hypothesis 2*. Pro-environmental beliefs and constructed beliefs about the risks and benefits associated with the construction of a uranium mine will influence the activation of emotional balance and subsequent acceptance of the mining proposal.

*Hypothesis 3*. The emotional balance activated by the proposal to construct a uranium mine will have a direct impact on the acceptance of the mining proposal while also mediating the relationships between perceived risks or benefits and the acceptance of the energy project.

## 2. Materials and methods

### 2.1. Nuclear energy project characteristics

**Figure 2:**
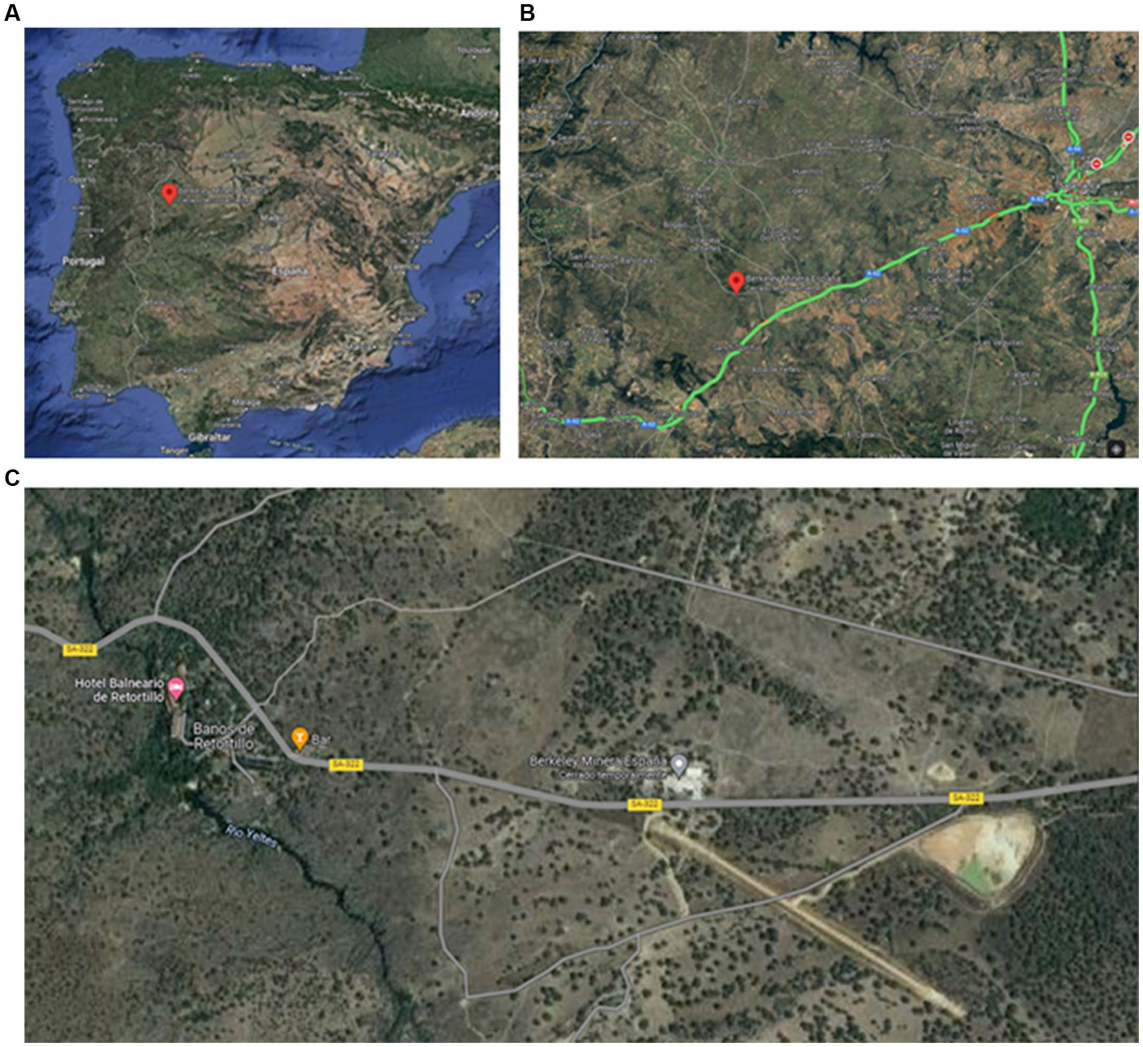
Geographical location of the uranium mine project called the “Salamanca Project” in Spain, which would become the largest open pit uranium mine in Europe. **(A)** The “Salamanca Project” is located in northwestern Spain, **(B)** it is located 70 kilometers from the city of Salamanca and 45 kilometers from the border with Portugal. It is an eminently agricultural area with a low population density in the nearby towns (Retortillo and Villavieja de Yeltes with 200 and 850 inhabitants, respectively); **(C)** The location of the mine is less than 2 kilometers from a thermal spa used by the Romans, in the area runs the Yeltes river. The image shows both the location of the tailings pond and the deforestation that has taken place in the area (the company claims to have already cut down more than 2,000 oak trees). Images extracted from google maps (for more information see https://bit.ly/3VIfRUA). Maps data: Google, ©2023 CNES/Airbus, IGP/DGRF, Maxar Technologies, Inst. Geogr. Nacional.

The uranium mine development and exploitation project, called the “Salamanca Project,” was planned to be carried out by an international private mining company in the province of Salamanca, in northwestern Spain (see Figure 2 for the exact geographic location of the uranium mine). Details of the Salamanca Project prepared by the mining company can be found on their website.^1^ The mining company began field studies in 2007 and intensified them in 2010, and from 2011 onward also began the social conflict and struggle against the project in the face of the approaches and actions in the mining field, creating different protest platforms, such as Stop Uranio.^2^ The project, which began in 2013, included a Retortillo-Santidad uranium mine, a uranium concentrates plant, and a radioactive waste storage facility. The concentrates plant is a radioactive facility and is subject to several authorizations, to be granted by the Ministry for Ecological Transition and Demographic Challenge, in accordance with a report from the Spanish Nuclear Safety Council. These authorizations are required for site selection, plant construction, commissioning, decommissioning, and dismantling. In July 2021, the project was rejected by the Spanish Nuclear Safety Council (CSN report, July 2021). However, the company appealed the decision and, at the time of this writing is awaiting the outcome of the appeal. Given the current energy crisis in Europe and the need to resort to energy from nuclear power plants, the debate has been reopened in the affected populations with conflicting opinions on the possible opening of the uranium mine to supply European and Spanish nuclear power plants.

### 2.2. Participants and procedure

By calculating an *a priori* power analysis with G*power software for correlation tests, analyses of variance (ANOVAs) for mean comparisons, and linear multiple regression as statistical tests, asking for a small to medium expected effect size, the optimal total sample size was 348 participants, with a power of 0.95.

A total of 371 people responded to the questionnaire we sent out. A portion of the sample (202 participants) was found by distributing the questionnaire to various places near the mine location using a snowball sampling method (Baltar and Brunet, 2012). The questionnaire was developed on the Google Forms platform, which could be accessed through a link and a QR code. To do this, we distributed the questionnaire in the affected area by placing posters with QR codes in different locations (bars, town hall, parks, and portals). To complete the sample to an optimal size, 169 university students from the University of Salamanca participated through a classroom activity in which participants could voluntarily access the questionnaire while respecting their anonymity. The ethical committee of the University of Salamanca approved the research design of this study (ref. 0000822, approved on November 24, 2022).

Regarding the sociodemographic characteristics of the sample, the 371 participants in the study had a mean age of 33.5 years (SD = 15.87, ranging in age from 18 to 80 years) with 63.1% women and 36.9% men. In terms of employment status, 43.7% reported working full-time, 50.1% were students, 3% were retired, 2.7% were unemployed, and 0.5% were in another situation. In terms of education level, 57.4% reported having university education, 13.7% had doctoral studies, 24% had high school studies, 4% had vocational training, and 0.8% had basic or elementary education. Regarding the monthly income level of the family unit, 31% reported earning more than 2,500 euros, 15.1% between 2001 and 2,500 euros, 15.1% between 1,501 and 2000 euros, 16.4% between 1,001 and 1,500 euros, 5.9% less than 1,000 euros, and 16.4% did not answer this question. They were also asked how far their place of residence was from the area affected by the uranium mine, with 59% reporting living less than 100 kilometers away, compared to 41% who reported living further away. 75.2% reported not having done tourism in the area affected by the mine, while 24.8% had. Finally, 17.5% claimed to have participated in some kind of protest activity against the mine, with only 1.3% saying they had participated in any action in defense of the mine’s construction. Descriptive analyses of the socio-demographic characteristics based on the origin of both samples can be found in the supplementary material (see Supplementary Material S1).

The questionnaire presented the following introduction:

We are a research group of the University of Salamanca that is conducting a survey on the social assessment of the uranium extraction and processing project for energy purposes in the province of Salamanca, specifically in the towns of Retortillo, Santidad and Villavieja de Yeltes. The participation in this survey is voluntary, so we would be grateful if you could answer the questions honestly, bearing in mind that we are interested in your assessment. There being no right or wrong answers. The information collected will be anonymous and will be used strictly for research purposes, so we understand that the completion of the test implies your consent to use the data for such purposes. The estimated response time is less than 15 min.

### 2.3. Measures

Immediately after the presentation, the questions related to sociodemographic variables, environmental beliefs, emotions about the mine, and personal assessment of acceptance were presented:

- *Sociodemographic and contextual measures.* Participants responded to questions related to age and sex; in addition, employment situation, socioeconomic status and educational level were assessed from a range with the five different options previously mentioned (5 being the highest level). Next, we asked about place of birth, place of residence, level of knowledge of the affected area and whether it was used as a tourist destination. The distance to the area affected by the mine project was calculated by asking whether the usual place of residence was located more or less than 100 km from the mine. Finally, we asked whether they had participated in actions to defend or reject the mining project.
- *Level of knowledge about the uranium mine exploitation project.* To assess whether individual perceived that they were sufficiently informed about the energy project (Ruiz et al., 2018), the level of knowledge of the population about both the uranium energy and the uranium mine project was evaluated with the next six specific items: In what measure do you think you have knowledge about… “the benefits of nuclear energy,” “the risks of nuclear energy,” “alternatives to nuclear energy,” “the project for the construction and operation of the uranium mine in Salamanca,” “benefits for the area of uranium mining as a nuclear energy source,” “the risks that the extraction of uranium as a source of nuclear energy would have for the region of Salamanca.” The questions were rated by participants using a 10-point Likert scale, with 1 = *no knowledge* and 10 = *high knowledge*. An exploratory factorial analysis with the six items showed one main factor which explained a 79.49% of variance. The scale had adequate reliability (*α* = 0.94).
- *Environmental beliefs.* The scale developed by Corral-Verdugo et al. (2008) and adapted to Spanish language by Ruiz et al. (2018) was used to assess the environmental beliefs of the population, including questions that consider both that *nature is at the service of humans* (e.g., “Caring for nature now means securing the future for humans”) and that *humans should take care of nature* (e.g., “Human beings can progress only by conserving nature’s resources”). The scale is composed of five items to which participants responded using a 10-point Likert scale, with 1 = *completely disagree* and 10 = *completely agre*e. An exploratory factorial analysis with the five items showed one main factor which explained a 61.09% of variance. The scale had adequate reliability in both the original (*α* = 0.78), and the present study (*α* = 0.84).
- *Perception of the benefits of the development of the uranium mine exploitation project*. We used an adaptation of the scale developed by Ruiz et al. (2018) to assess the extent to which participants perceived a benefit from the construction and exploitation project of the area-specific energy proposal. The scale consists of four items (e.g., “Uranium mining will help create new employment opportunities in Retortillo and the western part of the province of Salamanca”) to which participants are asked to respond on a 10-point Likert scale, with 1 = *completely disagree* and 10 = *completely agree*. The four items had referred to job opportunities, economic and political influence, economic investments, and social development. An exploratory factorial analysis with the four items showed one main factor which explained an 83.82% of variance. The scale had adequate reliability in both the original (*α* = 0.94) and the present study (*α* = 0.94).
- *Risk perception of the uranium mine development project*. We used an adaptation of the scale developed by Bronfman et al. (2012), which consists of four items (e.g., “Uranium mine developments pose a risk to human health”) to which participants are asked to respond on a 10-point Likert scale, with 1 = *strongly disagree* and 10 = *strongly agree*. The four items refer to health risk, a traditional economy, tourism activities, and environmental risk in the form of deforestation and water contamination. An exploratory factorial analysis with the four items showed one main factor which explained a 79.22% of variance The scale had adequate reliability in both the original (*α* = 0.92) and the present study (*α* = 0.91).
- *Emotional balance toward the uranium mine development project*. We used the reduced version of the Positive Affect and Negative Affect scale (Watson et al., 1988; used by Cuadrado and Tabernero, 2015), composed of eight items that are rated on a 10-point scale on which 1 = *strongly disagree* to 10 = *strongly agree*. Six items assessed negative emotional states activated by knowledge of the development a uranium mine project (annoyed, hostile, ashamed, fearful, nervous, and alert) and two items assess positive emotional states (confident and happy). An exploratory factorial analysis showed two main factors which explained a 76% of variance (55.63% for the 6 negative emotions and 21.71% for two positive emotions). The eight items showed an adequate reliability (*α* = 0.86). A global measure of emotional balance was created from the person’s mean negative affect was subtracted from the person’s mean positive affect (Wiese et al., 2000).
- *Level of acceptance of the development a uranium mine.* Acceptance was assessed by measuring the degree to which the participants agree with the proposed energy intervention, along the same lines as the work of Ruiz et al. (2018); specifically, they were expressly asked, in four items, about “To what extent do you accept a uranium mine development … in the Retortillo-Santidad area, your residence area, Spain, or other countries?.” Responses were made on a 10-point Likert-type scale (1 = *strongly disagree*, 10 = *strongly agree*). An exploratory factorial analysis with the four items showed one main factor which explained a 73.55% of variance. The scale had adequate reliability (*α* = 0.88).

### 2.4. Statistical analysis

We conducted descriptive and correlational analyses to test the relationships between the variables. Several ANOVAs were performed to test gender differences in the psychosocial variables incorporated. To test an exploratory model of the acceptance of a project of a uranium mine with all of the study’s variables, we performed structural equation modeling (SEM). A multigroup SEM analysis was conducted to test for the equivalence of the theoretical explanatory model structure among both samples. This was performed with IBM SPSS Statistics Amos (Version 25) by following the manual multistep proposed by Byrne (2009). The model fit was evaluated using the following statistics: χ^2^, χ^2^/*df* ratio, root-mean-square error of approximation (RMSEA), goodness-of-fit Index (GFI), adjusted GFI (AGFI), normed fit index (NFI), and comparative fit index (CFI). For model evaluation, Schermelleh-Engel et al.’s (2003) recommendations were followed: acceptable model fit is indicated by χ^2^/*df* ≤ 3; RMSEA <0.08, with a confidence interval (CI) close to RMSEA; GFI and NFI ≥ 0.90; AGFI between 0.85 and 0.90; CFI and Tucker–Lewis Index (TLI) ≥ 0.95; and Incremental Fit Index (IFI). Good model fit is indicated by χ^2^/*df* ≤ 2; RMSEA between 0 and 0.05, with CI close to RMSEA; GFI and NFI ≥ 0.95; AGFI higher than 0.90; CFI and TLI ≥ 0.97, and IFI. Finally, mediational analyses in IBM SPSS Amos (Version 25) were performed (Collier, 2020).

## 3. Results

### 3.1. Relationships between all studied variables

As a first step, Pearson correlation analyses was performed with the sociodemographic variables and the rest of variables evaluated in the questionnaire to test Hypothesis 1. The level of studies (*M* = 3.79, SD = 0.76) and income (*M* = 3.53, SD = 1.42) presented a significant and positive level of correlation with each other (*r* = 0.33, *p* < 0.001); in addition, both variables showed a significant level of correlation with age (*r* = 0.33, *p* < 0.001; *r* = 0.38, *p* < 0.001, respectively) and with the level of knowledge (*r* = 0.19, *p* < 0.001; *r* = 0.16, *p* = 0.008, respectively). On the other hand, education level showed a significant and negative relationship with both level of perceived benefits and level of acceptance with the proposed uranium mine construction (see Table 1).

**Table 1:** Means, standard deviations, and correlations between all the variables studied (bivariate Pearson correlations).

Variables	1	2	3	4	5	6	7	8
1. Age	–							
2. Academic level	0.34^**^	–						
3. Knowledge level	0.36^**^	0.19^**^	–					
4. Environmental beliefs	0.19^**^	0.10	0.12^*^	–				
5. Risk perception	−0.05	−0.01	0.11^*^	0.38^**^	–			
6. Benefits perception	−0.38^**^	−0.16^**^	−0.33^**^	−0.26^**^	−0.44^**^	–		
7. Emotional balance	−0.13^*^	0.04	−0.29^**^	−0.35^**^	−0.48^**^	0.48^**^	–	
8. Project acceptance	**−0.26** ^**^	**−0.11** ^*^	−0.09	**−0.29** ^**^	**−0.37** ^**^	**0.54** ^**^	**0.39** ^*^	–
*M*	35.53	3.79	3.98	9.09	7.80	5.41	−1.33	2.94
SD	15.87	0.76	2.30	2.47	2.10	2.43	2.99	1.94

^*^p < 0.05, ^**^p < 0.01.

The values in bold highlight the significant correlations between the variables studied and the dependent variable (project acceptance).

In regard to the role of gender, women presented significantly stronger environmental beliefs, *F*(1,369) = 8.23, *p* = 0.004; ŋ^2^ = 0.022; observed power (OP) = 0.816; *M* = 9.21, SD = 0.88, than men (*M* = 8.88, SD = 1.36), according with that difference women perceived a higher level of risk associated with the construction of the uranium mine, *F*(1, 369) = 6.59, *p* = 0.011; ŋ^2^ = 0.018; OP = 0.726; *M* = 8.01, SD = 1.85, than the level of risks perceived by men (*M* = 7.43, SD = 2.45), and therefore experienced stronger negative emotions, *F*(1,369) = 5.89, *p* = 0.016; ŋ^2^ = 0.016; OP = 0.678; *M* = 4.36, *SD* = 2.37; than the negative emotions experienced by men (*M* = 3.75, SD = 2.20). The emotional balance is significatively higher for women (*M* = −1.60, *SD* = 3.05; *F*(1,369) = 5.14, *p* = 0.024; ŋ^2^ = 0.014; OP = 0.619) than for male (*M* = −0.87, SD = 2.84), However, women reported having a lower level of knowledge about the uranium mine project, *F*(1,369) = 14.54, *p* = 0.001; ŋ^2^ = 0 0.038; OP = 0.967; *M* = 3.64, *SD* = 2.16, than the level of knowledge reported by men (*M* = 4.56, SD = 2.42). No significant differences were found with the rest of the variables analyzed, the level of agreement with the uranium mine was not significatively different between women and male (*F*(1,369) = 1.23, *p* = 0.267).

Regarding the differences related to access to the sample, the different ANOVAs performed between the overall sample and the student sample revealed significant differences in the following variables: environmental beliefs, *F*(1,369) = 10.56, *p* < 0.001; ŋ^2^ = 0.028; OP = 0.90; level of knowledge, *F*(1,369) = 50.40, *p* < 0.001; ŋ^2^ = 0.12; OP = 1.0; perception of benefits, *F*(1,369) = 53.68, *p* < 0.001; ŋ^2^ = 0.127; OP = 1.0; negative emotions, *F*(1,369) = 4.62, *p* < 0.05; ŋ^2^ = 0.012; OP = 0.57; and level of acceptance, *F*(1,369) = 43.74, *p* < 0.001; ŋ^2^ = 0.106; OP = 1.0. However, there were no significant differences in perceived level of risk, *F*(1,369) = 0.17, *p* = 0.68; ŋ^2^ = 0.00, OP = 0.069; and level of positive emotions activated against the construction of the uranium mine, *F*(1,369) = 0.10, *p* = 0.76; ŋ^2^ = 0.00; OP = 0.061. It should be noted that a significant difference was also found between the two samples with respect to age, *F*(1,369) = 26.77, *p* < 0.001; ŋ^2^ = 0.068; OP = 0.99, so the differences found may be due to this factor.

To evaluate the relationship between the other variables studied, we performed several correlation analyses. As can be seen in Table 1, all the relationships followed the expected direction (Hypotheses 1–3). Older participants showed a higher level of knowledge and academic level, and the more knowledge they claimed to have about energy from uranium mines, the lower level of the perception of benefits and the emotional balance towards uranium mining development. Environmental beliefs were shown to have a significant positive relationship with risk perception, although the relationship with perceived benefits, emotional balance, and level of acceptance of the project was significant and negative. Participants who reported a higher level of risk perception showed significantly and negatively fewer benefits, less emotional balance, and a lower level of acceptance of the uranium mine. Finally, the level of project acceptance showed negative significant correlations with environmental beliefs and risk perception, and positive significant correlations with benefit perception and emotional balance. These results allow us to test the theoretical model presented in Figure 1. The correlation table based on the origin of both samples can be found in the supplementary material (see Supplementary Material S2). The correlation analyses for both samples (general population and students) showed similar values between acceptance of the mining project and each of the analyzed psychosocial variables: environmental beliefs [−0.23/−0.29, respectively], risk perception [0.41/0.36, respectively], perception of benefits [−0.50/−0.43, respectively], positive emotions [0.36/0.34, respectively], and negative emotions [−0.17/−0.31, respectively]. Despite the differences in the sociodemographic characteristics of the participants according to the data collection carried out (general population versus university students), the values found in the correlation analyses and the significance levels were very similar, and therefore the statistical analyses were performed with the entire sample.

### 3.2. Structural equation model for the psychosocial variables explaining the level of acceptance of a uranium mine construction project

**Figure 3:**
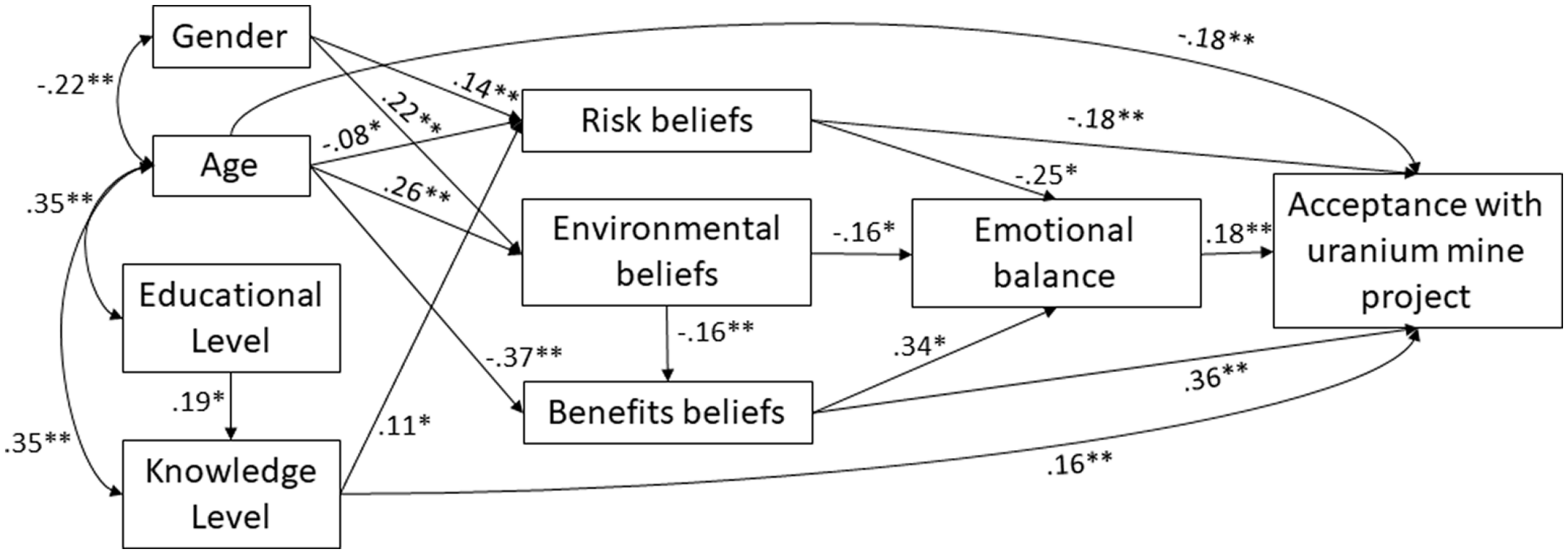
Structural equation modeling analysis to explain the level of acceptance of a uranium mine from sociodemographic, cognitive, and emotional variables involved in the personal assessment. Numeric values indicated the strength of the standardized estimates (*β*). All relationships signed between variables were significative at least at the 0.05 level.

To test all our hypotheses and the hypothesized theoretical model of the acceptance of the uranium mine (see Figure 1), we performed a SEM analysis, testing for the adequacy of the theoretical explanatory model structure. Figure 3 represents the validity of the theoretical model for the entire sample. The goodness-of-fit tests revealed that the model was well fitted, *χ*^2^(9) = 7.022, *p* = 0.635; CMIN/df = 0.780; RMSEA = 0.000 (95% CI [0.00, 0.049]); CFI = 1.00; NFI = 0.991; IFI = 1.00; NFI = 0.991.

**Figure 4:**
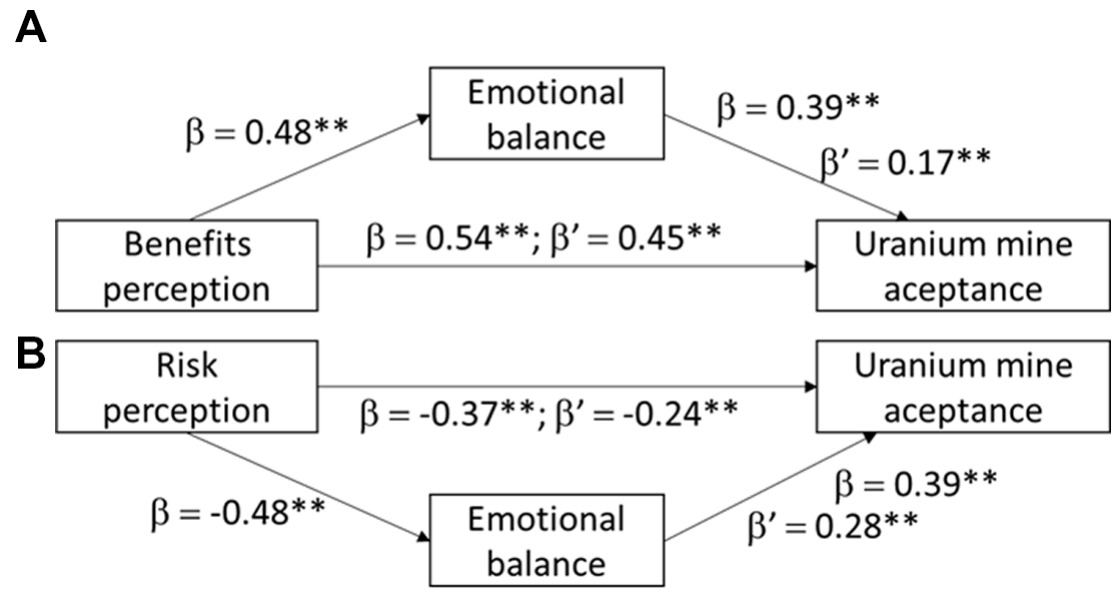
Mediational analysis of emotional balance activated before the construction project of a uranium mine in the relationships between **(A)** benefits perception of the mine with acceptance of the mine and between **(B)** risk perception with acceptance of the mine (**p < 0.01).

To test Hypothesis 3, two simple mediational models had been created based on the emotional balance relationships tested in the path analysis and agreed with the theoretical model shown in Figure 3. The emotional balanced aroused by the creation of the uranium mine had mediated the relationship between the perceived of benefits (see Figure 4A) and risk of the mine (see Figure 4B). To test mediation, 3 pathways were examined: (1) the main chain leading from the level of perceived benefits or risks (Figures 4A,B) of mine construction (as independent variables, IV) to the level of acceptance of the uranium mine project (as dependent variable, DV); (2) the simple mediation pathway through emotional balance (mediator, M) to mine acceptance (DV); and (3) the simple mediation pathway from the level of perceived benefits or risks of mine construction (IV) through emotional balance (M) to mine acceptance (DV). Confidence intervals (95%) were generated by bootstrapping with 5,000 resamples. Bootstrapping is a nonparametric resampling procedure that allows confidence intervals (CI) to be generated for statistical inference when normality assumptions about the sample distribution are not required.

The study assessed the mediational role of emotional balance on the relationship between benefits perception (and risk perception) and the level of acceptance of the uranium mine. The results revealed a significant indirect effect of impact of emotional balance on the relationship between benefits and level of uranium mine acceptance was positive and significant (indirect effect = 0.219; *p* < 0.001, [lower confident Interval = 0.037; bound confident Interval = 0.099]), the indirect effect of the impact of emotional balance on the relationship to risk was also significant, supporting H3. Furthermore, the direct effect of benefits perception on uranium mine acceptance in presence of the mediator was also found significant (both when considering benefits *β*′ = 0.45**; and considering risks *β*′ = −0.24**). Therefore, emotional balance partially mediated both the relationship between benefits and level of acceptance and the relationship between risks and level of acceptance of the uranium mine.

## 4. Discussion and conclusions

The rejection of mine construction is not something new in the history of mineral extraction; being aware of this, Ey et al. (2017) stated that acceptance by or rejection of a community in response to the construction of a mine is far from being a rational decision but obeys other factors that have gone relatively unnoticed, such as psychosocial variables. Devine-Wright and Wiersma (2020) stated that acceptance is a multidimensional concept that integrates social, psychological, affective, historical, demographic, economic, and political variables. The result of our research has aimed to show this evidence through an explanatory model of the acceptance of the proposal of a uranium mine after taking into consideration the interrelation of sociodemographic and contextual variables (e.g., age, gender, economic and education level, perceived level of knowledge), cognitive variables (e.g., pro-environmental beliefs, perception of risks and benefits), together with emotional balance (as an equilibrium between the positive and negative emotions), that are activated by the proposal of the mine construction.

In line with previous research results (Devine-Wright and Batel, 2013; Bjørst, 2016), sociodemographic characteristics played an important role in explaining the acceptance or rejection of the uranium mine. As expected, participants with higher education levels had greater knowledge of the project. Our study confirmed that older participants were less accepting of the uranium mine project, although they had more knowledge about it and nuclear energy, consistent with the findings of Pedersen and Johansson (2012). In contrast, Pedersen and Johansson (2012) found a positive relationship between age and acceptance of a wind farm, but a negative and significant relationship between environmental beliefs and uranium drilling. Our study highlights the relevance of environmental beliefs and emotions towards a uranium extraction project associated with nuclear power and its potential acceptance. We found that older people perceived greater risks and fewer benefits from the development of the mining project, which was consistent with pro-environmental beliefs. Our results also support the findings of other research (Flynn et al., 1994; Gao et al., 2022) that women held stronger pro-environmental beliefs, perceived greater risks towards the mining project, and expressed more negative emotions. Flynn et al. (1994) found that differences regarding perceived risk decrease when considering the interaction of gender with the racial origin of the sample (white or non-white). Based on our data, we found that gender differences in perceived risk became non-significant when we analyzed only the sample of those under 30 years of age (60% of the sample, from *F*(1,369) = 6.59, *p* < 0.01 to *F*(1,230) = 3.01, *p* = 0.08). This result suggests the need to address gender differences in interaction with age when analyzing the valuation of new energy projects, whether they are renewable energy projects, such as wind energy, or based on nuclear energy development.

Furthermore, the results showed that individuals with higher education levels perceive fewer benefits from the development of the uranium mine construction project and, therefore, have lower acceptance of the project. This result is consistent with Brannstrom et al.’s (2022) proposal that, in isolated communities with few development opportunities, the perception of benefits of the mining project and the consequent acceptance grow in people with low education levels and low job expectations. Along these lines, Corner et al. (2011) created a sociodemographic profile of people who would presumably be in favor of nuclear energy as male, older, and of a high social class, whereas pro-environmental values and beliefs would act as more stable predictors of nuclear energy rejection. Thus, individuals with strong pro-environmental beliefs or values were less likely to approve uranium mining or nuclear power projects as a way to combat climate change, although acceptance or rejection was mediated by the perceived risk of the project (Corner et al., 2011). In contrast to Corner et al.’s (2011) results on the greater acceptance of the mine as a function of age, the explanatory model presented found that older people hold both greater pro-environmental beliefs and higher levels of information but fewer perceived benefits and less acceptance. Thus, according to the results found by Devine-Wright (2009), the analysis of the acceptance of a proposal for the construction of a mine should always take into account the characteristics of the sample, so that the theoretical model of interrelation between variables should consider the sociodemographic characteristics of the population in the explanatory model, such as age, gender, educational level, or level of knowledge on the subject. By taking these factors into account, it may be possible to increase the likelihood of acceptance and reduce potential conflicts in the communities affected by energy projects.

According to the path analysis created, the emotional balance would have a partial mediation role both between the perception of risk and the level of acceptance of the uranium mine proposal, as well as with the perception of benefit and the level of acceptance of the uranium mine. These results are in line with those found by Ransan-Cooper et al. (2018), who stated that in the face of fear of an energy project, negative emotions are activated that explain rejection and, in many cases, it is these emotions that agglutinate the rejection of the citizens that make up the affected populations (Johansson et al., 2022). Therefore, in the proposed theoretical model we present the emotional balance as a mediator in the acceptance or rejection of a proposal in such a way that when the perceived risk is high, negative emotions increase, therefore the emotional balance becomes negative and the impact of the perceived risk on the probability of accepting the mining proposal decreases. When the perception of a possible benefit from the mine increases and therefore increases the probability of acceptance, the emotional balance would act as a mediator, reducing the direct impact of this relationship.

Our results are in line with the hypothesis proposed by Bronfman et al. (2012), according to which the perception of risks and benefits of the proposed renewable energy sources explained social acceptance by a local community, where the perception of benefits had the greatest influence on acceptance. Thus, in the results shown in the path analysis, the variable with the greatest explanatory power for acceptance was the perceived benefits of uranium mine construction. However, this impact was mediated by the activation of emotional balance in reaction to the development of the uranium mine. Overall, the present study highlights the importance of considering emotional responses when examining the acceptance or rejection of proposed energy projects. It suggests that emotional balance plays a crucial role in mediating the relationship between perceived risks and benefits and the level of acceptance. Therefore, it is essential to address the emotional responses of affected populations and take into account their concerns and fears when proposing new energy projects. This approach can help foster greater understanding and collaboration between local communities, policymakers, and energy companies, leading to more sustainable and socially acceptable energy projects.

This research has some limitations. One is centered on the methodological approach we adopted; a micro sociopsychological vision was used to analyze how the psychosocial variables influence acceptance of a new energy proposal, but the interrelation with more macro-level variables associated with policies, institutions, or economic markets was left aside, as Devine-Wright and Wiersma (2020) suggested. On the other hand, another limitation comes from the procedure used to access the sample, a part of the participants in the study came from a data collection based on a snowball system distributed in the area affected by the construction of the mine, while another part of the sample were students from a university close to the area affected by the mine. Another limitation was that participants’ attachment to place, affinity or identity with the affected area was not assessed, which would have allowed us to analyze differentiation in beliefs about risks and benefits, as well as emotional balance and acceptance level. Nor did we evaluate trust or mistrust of the project development and institutions, another variable that has been found to be relevant in other studies on mine construction (Lehtonen et al., 2022). On the other hand, the data were collected at a single point in time without considering the evolution of the conflict in the area. As mentioned previously, the proposal for the construction of the mine started years ago and is currently under appeal by the company. It would have been interesting to conduct a longitudinal follow-up of the social construction created toward the project, both in terms of attitudes, the level of information, the balance between risks and benefits and between positive and negative emotions activated by the mining project over time.

As implications for environmental management, the results of this study highlight the need to analyze the emotions citizens feel when faced with energy project proposals, especially for people with ties to the affected area. The emotional balance moderates the possible benefits or intensifies the perceived risks. Given the interrelationships among the variables, the greater the perception of the benefits associated with the development of the uranium mine, the greater the positive emotions generated and the greater the probability of acceptance of the project. Although the perception of risk intensifies negative emotions, we believe that this relationship will be determinant of a low acceptance of the proposed construction of the uranium mine, especially among citizens related to the affected area. Furthermore, the results have shown the relevance of environmental beliefs, which were negatively related to perceived benefits, emotional balance and acceptance of the mine construction while presenting a positive relationship with perceived risks; therefore, the preservation of nature seems to be present before making the decision to accept or reject a uranium mining project. The results should be considered to manage and prevent the social conflicts generated among the populations affected by the construction of new mining projects. When dealing with a uranium mine construction project, the evaluation of the activation of negative emotions takes on special attention for the management of conflicts both between communities and between citizens. Furthermore, the communication media should be appealed by this issue since they have the responsibility to provide rigorous information to their audience. When dealing with this kind of interventions, the media, local stakeholders, and municipalities should cover in an objective way the implications of the projects in all the different levels, without aiming to bias the perceptions of citizens by favoring dialogue rather than conflict over the diversity of perceived risks and benefits.

## Data availability statement

The raw data supporting the conclusions of this article will be made available by the authors, without undue reservation.

## Ethics statement

The studies involving human participants were reviewed and approved by the ethical committee of the University of Salamanca approved the research design for this study (ref. 0000822, approved on November 24, 2022). Written informed consent for participation was not required for this study in accordance with the national legislation and the institutional requirements.

## Author contributions

GS-T, AH, JG, and CT contributed to conception and design of the study, and wrote sections of the manuscript. GS-T organized the database. GS-T, AH-M, and CT performed the statistical analysis. GS-T and CT wrote the first draft of the manuscript. All authors contributed to the article and approved the submitted version.

## Funding

This research was supported by the Spanish Ministry of Science and Innovation (NextGenerationEU and Recovery Transformation and Resilience Plan) under grant number TED2021-130924B-100, in which CT and AH are the principal investigators.

## Conflict of interest

The authors declare that the research was conducted in the absence of any commercial or financial relationships that could be construed as a potential conflict of interest.

## Publisher’s note

All claims expressed in this article are solely those of the authors and do not necessarily represent those of their affiliated organizations, or those of the publisher, the editors and the reviewers. Any product that may be evaluated in this article, or claim that may be made by its manufacturer, is not guaranteed or endorsed by the publisher.

## Footnotes

^1^ www.berkeleyenergia.com/salamanca-project-overview/

^2^ https://twitter.com/stopuranio?lang=es
