# Supplementary material for: Explanatory model of the psychosocial variables related to the social acceptance of a uranium mine project in northwest Spain
Source: Front Psychol. 2023 May 23;14:1134499. doi: 10.3389/fpsyg.2023.1134499 (PMC10242019; doi:10.3389/fpsyg.2023.1134499)
Supplement: Supplementary file 1 [file Data_Sheet_1.PDF]

S1. Supplementary material

Table 1. *Descriptive characteristics of the local and student samples and statistical analyses to compare the distributions.*

**Variables**	**Local sample (n = 202)**	**Student sample (n=169)**	**Statistical differences**
Age	Mean = 43.63 (Sd = 15.36)<br>Range: 18 to 80	21.46 (Sd = 1.78)<br>Range: 19 to 33	F (1,369) = 348.09<br>$p < .001$
Gender	52% male / 48% female	19% male / 81% female	$\chi2$ (DF=1) = 43.14<br>$p < .001$
Employment status	79.7 % full time job<br>9.90 % student<br>5.4% retired<br>4% unemployed<br>1% other situations	0.6 % part time job<br>98.2 % student<br>1.2 % unemployed	$\chi2$ (DF=4) = 288.58<br>$p < .001$
Educational status	59.4% university studies<br>25.2% doctoral studies<br>8.4% bachelor studies<br>5.4% professional training<br>1.5% elementary studies	55% university studies<br>42.6 % bachelor studies<br>2.4% professional training	$\chi2$ (DF=4) = 92.47<br>$p < .001$
Economic status	1 (less than 1000E) - 4.5 %<br>2 (1001 to 1500E) – 18.3%<br>3 (1501 to 2000E) – 11.9%<br>4 (2001 to 2500E) – 11.4%<br>5 (more than 2500E) -41.1%<br>Non answered – 12.9%	1 – 7.7 %<br>2 – 14.2 %<br>3 – 18.9 %<br>4 – 0 %<br>5 – 18.9 %<br>Non answered – 20.7%	$\chi2$ (DF=5) = 27.66<br>$p < .001$
Kilometers from actual residence	64.5% less than 100 km<br>35.6% more than 100 km	52.7% less than 100 km<br>47.3% more than 100km	$\chi2$ (DF=1) = 5.20<br>$p < .05$
In-deep knowledge	87.6% yes<br>12.4% no	20.7% yes<br>79.3% no	$\chi2$ (DF=1) = 168.23<br>$p < .001$
Tourism in the area	42.6% yes<br>57.4% no	3.6% yes<br>96.4% no	$\chi2$ (DF=1) = 75.14<br>$p < .001$
Active acts for project defense	2.5 % yes<br>97 % no<br>0.5% non-answered	0% yes<br>100 % no	$\chi2$ (DF=1) = 4.26<br>$p < .05$
Active acts for project rejection	69.3 % yes<br>30.7 % no	1.8 % yes<br>98.2 % no	$\chi2$ (DF=1) = 53.25<br>$p < .001$

S2. Supplementary material

Table 2. *Means, standard deviations, and correlations between all the variables studied (bivariate Pearson correlations).*

**Variable**	**1**	**2**	**3**	**4**	**5**	**6**	**7**	**8**	***M*** n=169	***SD***
1.Age	—	-0.08	0.16*	-0.02	-0.08	-0.03	-0.05	-0.09	21.46	1.78
2.Knowledge level	0.21**	—	-0.03	0.22**	-0.14	0.02	0.33**	-0.15	3.11	1.47
3.Environmental beliefs	0.12	0.12	—	0.31**	-0.01	-0.19*	0.30**	**-0.29****	8.89	1.00
4.Risk perception	-0.10	0.08	0.49**	—	-0.17*	-0.28**	0.38**	**-0.36****	7.75	1.58
5.Perception of benefits	-0.21**	-0.26**	-0.31**	-0.55**	—	0.21**	-0.14	**0.43****	6.36	1.50
6.Positive emotions	-0.11	0.24**	-0.13	-0.20**	0.28**	—	-0.06	**0.34****	2.84	1.97
7.Negative emotions	0.05	0.55**	0.31**	0.45**	-0.50**	0.07	—	**-0.31****	3.85	1.82
8.Project acceptance	-0.05	0.10	**-0.23****	**-0.41****	**0.50****	**0.36****	**-0.17***	—	3.64	1.59
M (local, *n* = 202)	43.63	4.71	9.25	7.84	4.62	2.78	4.37	2.37	—	—
SD	15.36	2.60	1.14	2.47	2.76	1.90	2.65	2.02	—	—

Note. The values under the diagonal belong to the local sample. The values on the diagonal belong to the sample of students (*$p < 0.05$, **$p < 0.01$).
